# Human influences shape the first spatially explicit national estimate of urban unowned cat abundance

**DOI:** 10.1038/s41598-021-99298-6

**Published:** 2021-10-28

**Authors:** Jennifer L. McDonald, Elizabeth Skillings

**Affiliations:** 1 Veterinary Department, Cats Protection, National Cat Centre, Haywards Heath, RH17 7TT UK; 2 Bristol Veterinary School, University of Bristol, Bristol, BS40 5DU UK

**Keywords:** Ecology, Population dynamics, Urban ecology, Ecology, Population dynamics, Urban ecology

## Abstract

Globally, unowned cats are a common element of urban landscapes, and the focus of diverse fields of study due to welfare, conservation and public health concerns. However, their abundance and distribution are poorly understood at large spatial scales. Here, we use an Integrated Abundance Model to counter biases that are inherent in public records of unowned cat sightings to assess important drivers of their abundance from 162 sites across five urban towns and cities in England. We demonstrate that deprivation indices and human population densities contribute to the number of unowned cats. We provide the first spatially explicit estimates of expected distributions and abundance of unowned cats across a national scale and estimate the total UK urban unowned cat population to be 247,429 (95% credible interval: 157,153 to 365,793). Our results provide a new baseline and approach for studies on unowned cats and links to the importance of human-mediated effects.

## Introduction

Globally, the domestic cat (*Felis catus*) is one of the most ubiquitous mammals. In the UK, there are more than 10 million owned cats^1,2^, however they only make up a subgroup of the total population with many domestic cats unowned. Despite an understanding of the scale of the unowned cat population within UK shelters (in excess of 130,000 in shelters each year^3,4^), there are currently no evidence-based figures for the abundance or density of unowned free-roaming cat populations. These cats may be stray cats (abandoned or lost cats that were previously owned) or unsocialised feral cats, which, when unneutered and given access to appropriate resources, can be extremely prolific breeders. Unlike owned cats, which are primarily managed by private individuals, unowned cats are often living uncontrolled receiving limited to no intentional human support. The concerns surrounding these populations are well-documented and span multiple fields of research including animal welfare (poor health^5,6^, high mortality^7,8^, anthropogenic threats ^9,10^), conservation (predation^11^, competition^12^ and disease transmission^12^) and public health (zoonotic diseases^13,14^). Despite the well-recognised interest in unowned cat populations, there is a lack of understanding of their abundance at broad spatial scales.

To date knowledge on unowned cat abundance has either been limited to local sites^9,15–18^ or largely inferred from our understanding of free-ranging cats due to difficulties determining whether a cat is owned or unowned^18–20^. This is especially true for countries, like the UK, where the majority of owned cats are routinely provided outdoor access^21^, and a particular challenge in urban areas, which both unowned and owned cats cohabit at higher densities than their conspecifics in rural areas^22^. Sampling protocols developed for use in rural areas may not be easily implemented in built up areas where a lack of accessibility to private areas behind homes and businesses can pose further logistical difficulties. A citizen science approach is well suited to urban areas enabling data collection from areas that field-scientists would be unable to access^23,24^. However, the potential for bias from publicly collected data can make the validity of predictions obtained from them questionable. Therefore, engagement with communities for widespread reporting of unowned cats needs to be paired with appropriate data validation that can account for biases in citizen science datasets.

To address these challenges, we provide an alternative approach using an Integrated Abundance Model (IAM). This model accounts for both the underdetection of unowned cats and misidentification of owned cats as unowned by integrating data from residents with expert data that apply robust protocols that more accurately identifies an unowned cat with no risk of double counting^25^. Integrating citizen science data with expert data of higher quality from a subset of sites within IAMs has been shown to produce model parameter estimates that are both precise and accurate^25^. Applying this model framework to data collected across a broad range of urban areas allows both the quantification of the unowned cat population and importantly the identification of conditions that enable populations to persist. In urban areas, unowned domestic cats live alongside people, thus various societal, demographic and environmental characteristics of urban areas have the potential to affect and consequently predict the abundance of unowned cats. By identifying properties of areas that are more likely to maintain unowned cat populations, it will be possible to derive evidence-based abundance estimates across different spatial scales.

To this end, we present a successful application of an IAM to citizen science data, paired with expert data on unowned cat abundance across five urban towns and cities in the UK. We find that the density of unowned cat populations is greater in areas that are more deprived, as well as for areas that have higher human population densities. We build off these results to predict distribution maps of abundance across urban areas and derive a first evidence-based estimate of total unowned cat abundance for urban areas across England and the UK. These results can be used to improve the monitoring of this previously intractable subgroup and allow appropriate and spatially efficient management to serve to improve their welfare and reduce numbers. Additionally, our results underscore the need to address social and economic conditions that support higher densities of unowned cats.

## Results

### Unowned cat abundance across five urban areas

**Figure 1:**
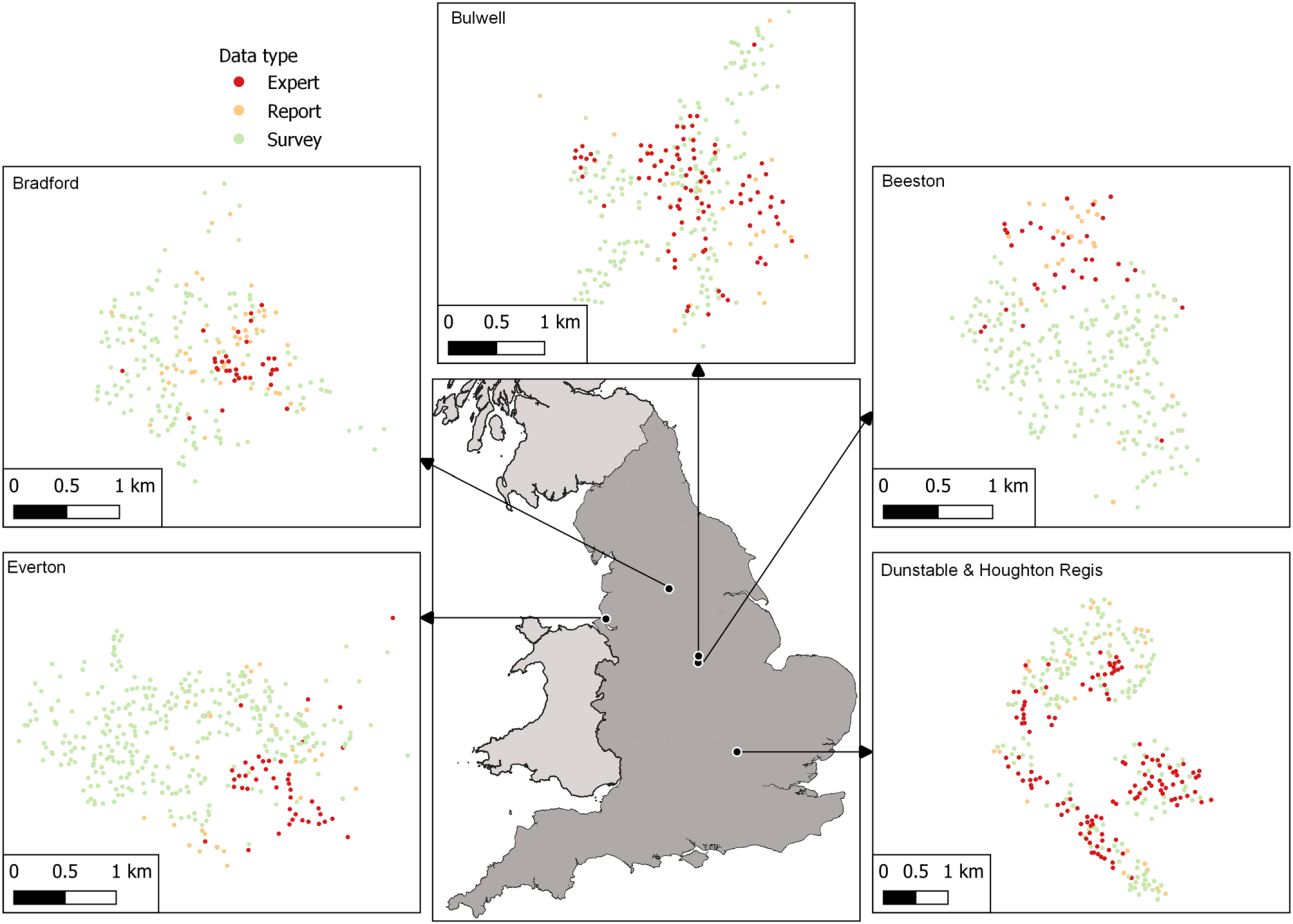
The location of our unowned cat records. Five key study areas in England and geographical distribution of data types within the study regions. Map was generated using QGIS 3.16 (https://www.qgis.org).

Within an IAM we analysed 877 resident reports, 3101 cross-sectional survey responses and 601 expert reports across 162 sites (each site encompasses a cluster of records within 500 m of each other, see “Data collation and preparation” in the “Methods” section) within five urban areas comprised of cities and towns in the UK (Fig. 1). The areas spanned a range of characteristics (Supplementary Table 1).

Expert data covered 64% of sites (104/162), which allowed inference of the error rates from the two citizen-science observer types (resident reports and door-to-door surveys), providing estimates of population abundance across all sites.

### Area characteristics and associations with unowned cat abundance

To explore the variation of unowned cat abundance among the 162 sites within five urban areas, we tested four possible types of influences. (1) Socio-economic context of the area according to English indices of multiple deprivation (IMD, the decile group 1 represents the least deprived 10% of residents in England and group 10 indicates the most deprived 10%). (2) Human population density, which will also be confounded with the owned cat population therefore, in the absence of fine-scale spatial data on the abundance of owned cats with outdoor access, this measure also acts also as a proxy for the number of owned cats within an area. (3) Indicators of the physical environment (dominant housing type, proportion of housing that are flats, type of urban settlement according to ONS classifications). (4) The proportion of usual residential population by ethnicity according to the broad headings used in the 2011 census where proportions differed by more than 70% across sites to provide an adequate range of testable variability. See Supplementary Table 1 for area characteristics and Supplementary Table 2 for resources used in this analysis. With known difficulties in model selection within hierarchical Bayesian frameworks, we used generalised linear models to explore the significance of each variable in relation to site-specific abundance estimates derived from the null IAM with just a random effects term. Significant variables were built into the IAM framework for further testing and to derive posterior probabilities of effect-sizes. Additionally, validation through simulations with known parameter values and the data structured similar to the raw data confirmed the accuracy of our IAM approach to detect covariates (Supplementary Figs. 1).

Across resident reports, there was 24.51% (20.57 to 28.89; 95% CRI) probability of detecting an unowned cat when present and on average 1.8 (1.58 to 1.94) owned cats were misidentified as unowned at each site. Across surveys, there was 17.92% (15.63 to 20.5; 95% CRI) probability of detecting an unowned cat when present and on average 0.72 (0.67 to 0.77) owned cats were misidentified as unowned at each site.

**Figure 2:**
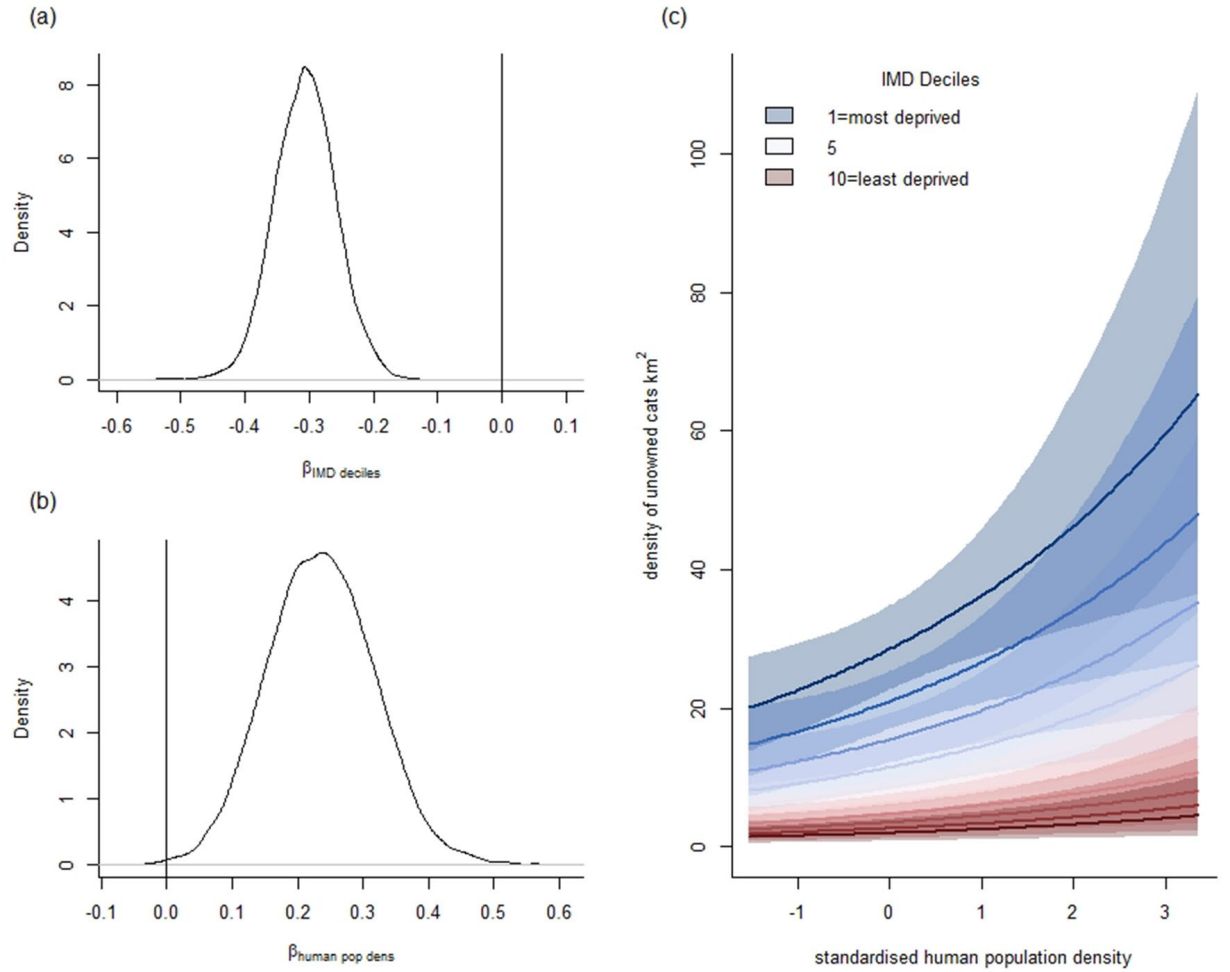
The influence of IMD deciles and standardised human population density on unowned cat abundance. (**a**,**b**) show the corresponding posterior distributions of the regression coefficients from an IAM (**c**) showing the predicted relationship and the corresponding 95% CRI between density of unowned cats and covariates.

Our results show two significant drivers of the abundance of unowned cats. The results for all model covariates are given in Supplementary Table 1. First, unowned cat abundance increased in areas that were more deprived (Fig. 2). Second, unowned cat abundance increased with human population density (Fig. 2). The posterior probability (our model-based belief) of increased unowned cat abundance with deprivation (i.e. probability β_IMD deciles_ < 0)) is 100% and of increased cat abundance with population density (i.e. probability β_human_ _pop_ _dens_ > 0)) is 99.9%. These variables explained 37% of variation in unowned cat abundance. Assessment of the effect of IMD and population density in separate model runs found IMD explained 31% of the variation in unowned cat abundance, whereas population density explained 7% variation in unowned cat abundance, the posterior probabilities of their effect were unchanged.

Our IAM applied to field data were robust against general Bayesian checking procedures, with convergence reached for all parameters and none of the investigated prior variants compromised our inferences (Supplementary Fig. 2). Further model validation found estimated effect sizes were robust against any variation across study sites with comparable results across cross-validated model predictions where the model was rerun with each study area withheld (Supplementary Fig. 3). Additionally, predictions were still informative where one form of citizen science data was withheld from the model, although reports alone were unable to infer on human population density due to reduced spatial coverage (Supplementary Fig. 4). Finally, analysing the data within a general linear model, that has known model-checking procedures found similar effect sizes that lie within the credible intervals of the IAM output (GLM β_IMD deciles_ = −0.32; β_human pop dens_ = 0.31).

### Inferring distribution and abundance of unowned cats in urban areas in England

To derive an estimate for unowned cats in urban areas in England we combined our validated model-derived posterior distributions for effect sizes with spatial statistics on deprivation and population density. We base our estimates on lower super output areas (LSOAs), the small area geographies for which there are national statistics available on deprivation and population density.

Human population densities observed in the study sites were generally representative of urban LSOAs in England (mean = 4903 vs 5,303 people per km^2^ respectively). However, 4.6% (n = 1257/27,246) of urban LSOAs in England had higher human population densities than that observed in the study. Consequently, we truncated the effect size of population density to the upper limit of the densities studied thus providing a conservative estimate based on inference from our findings only.

By truncating the effect of human population density, we prevent over inflation in areas with densities not explored in our study, including areas in London. We cannot discount that patterns of unowned cats are significantly different in London, with cat ownership thought to be lower^26^. However, the inclusion of London areas in our estimates did not informatively change findings as evidenced by overlap of the 95% CRIs (see Supplementary Table 3), thus full results are presented here.

Focusing on urban areas in England that cover approximately 24,823 km^2^ of land area, we estimate the population size of unowned cats to be between 118,391 to 258,882 with 95% credibility (mean = 179,494). Our estimate of unowned cats was robust to the effect sizes derived from different modelling approaches (Supplementary Table 3).

### Inferring distribution and abundance of unowned cats in urban areas in the UK

Small area geographies differ between countries in the UK and are defined by their respective national statistics agencies. Consequently, areas were only included if they were part of settlements of more than 10,000 people (see “Quantification of unowned cat abundance in UK” in the “Methods” section), this covered approximately 13% of the UK (Table 1).

**Table 1:** Country-specific estimates of unowned cats and their 95% credible intervals, alongside a combined UK measure.

	Area (size of urban area covered)	Mean unowned cat Population	Lower 95% Credible Interval	Upper 95% Credible interval
Country-specific	England (24,823 km^2^)	193,698*	122,479	287,608
	N. Ireland (920 km^2^)	11,068	7,370	15,699
	Scotland (2,739 km^2^)	21,218	13,418	31,418
	Wales (2,242km^2^)	21,445	13,886	31,068
Total	UK (30,724 km^2^)	247,429	157,153	365,793

*Estimates for England here are estimated using the standardised IMD quintiles to allow UK-wide estimates and are consequently different, although informatively similar, to the estimates that used the deciles.

UK measures of socioeconomic deprivation differ across country borders and naively comparing across these measures can result in problems in analysis ^27^. Previous research has demonstrated and published a consistent measure of deprivation, in the form of adjusted IMD scores ^27^, based on 2011 indices. These are quintiles (1 = least deprived; 5 = most deprived) and were equally found to be significant as deciles (Supplementary Table 1). They had high credibility in the IAM (100% probability that Quintiles have an effect; Supplementary Fig. 5) however, the model explained less variation compared to the deciles (26% vs 37%). In the absence of a consistent IMD decile across countries, this adjusted measure provided the means to inform on unowned cat abundance across the UK.

We generated an estimated range of unowned cats in urban areas by applying the posterior distributions of effect sizes to IMD quintiles and population density for 33,988 urban small area geographies (Table 1). The estimated size (and 95% credible intervals) of the unowned cat population in urban areas across the UK was 247,429 (157,153 to 365,793; Table 1). Our estimate of unowned cats in England was robust to the choice of deprivation data as evidenced by an overlap in their credible intervals (Table 1).

**Figure 3:**
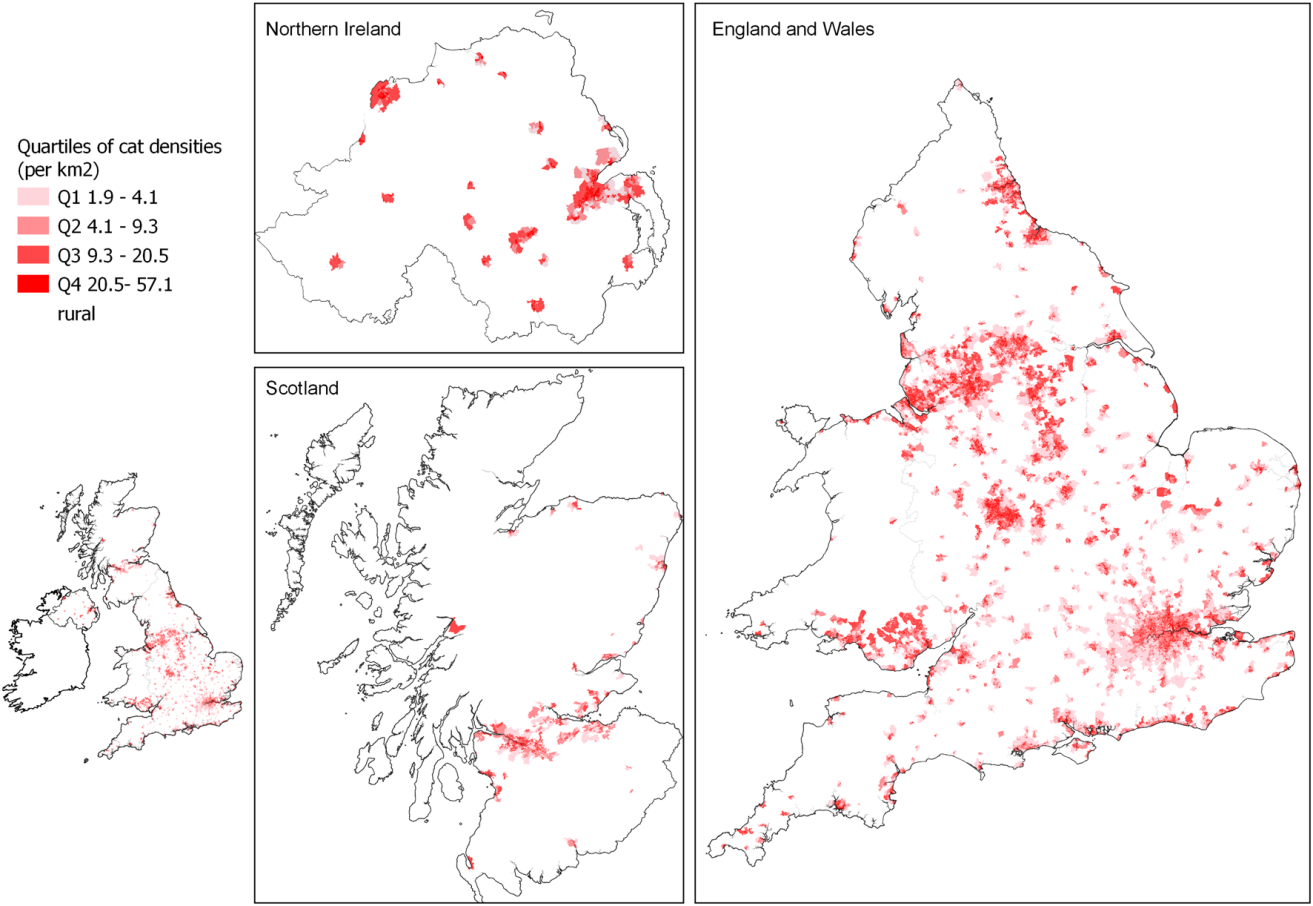
Density of unowned cats across urban areas in the UK. Density predicted from applying the posterior distributions of effect sizes derived from the IAM to IMD quintiles and population density for urban small area geographies. Mean values are shown. Countries are displayed separately according to their different urban geographical units. For visualisation purposes, some islands that do not include any urban areas are excluded from the plot. Map was generated using QGIS 3.16 (https://www.qgis.org).

At the finer spatial scale, we estimated a median cat density of 9.3 cats per km^2^, with site-specific averages ranging from 1.9 to 57 (Fig. 3).

In the absence of previous estimates of unowned cats in the UK, the predicted densities of unowned cats were validated at the scale of the LSOAs based on estimates from the IAM analysis of the raw data. There was a significant correlation between mean cat density per LSOA estimated from the IAM modelling applied to the raw data and the predicted densities (*p* = 0.65, p < 0.001). At LSOA-level we found 89% of predictions overlapped the 95% credible intervals for density estimates modelled from the raw data, there were no identifiable systematic bias across mean estimates (Supplementary Fig. 6). At the level of town and city credible intervals between model predictions and IAM outputs overlapped consistently and the average density across all study sites were quantitatively similar between the predicted and modelled raw data (Supplementary Fig. 7). Therefore, we acknowledge that estimates will be subject to error at fine spatial scales with further refinement and model development valuable. However, precision in estimates improves when aggregated to a larger area, validating the approach as a first estimate of abundance of UK unowned cat populations.

## Discussion

The presence of unowned cat populations in urban areas is often of concern due to poor animal welfare outcomes, zoonotic and non-zoonotic animal diseases and environmental impacts. However, their abundance and distribution on a national scale has been largely intractable to date, in part due to challenges with accurate identification of an unowned cat and a lack of accessibility in built-up areas. Here we extend an IAM to estimate drivers of unowned cat abundance. Our approach utilises the advantages of citizen science data, including wide scale reporting and accessibility, but controls for several sources of bias. Specifically, we account for duplicate reporting of closely situated residents, and the inclusion of expert data counters any bias in observations due to incomplete detection and misidentification of owned cats as unowned. In doing so, this study identified broad scale causes of unowned cat abundance to provide the first empirical estimate of expected distributions and abundance of unowned cats at a national scale. These estimates are critically informative as demonstrated through model validation. In addition, because unowned cat abundance in urban areas reflects fundamental properties of human communities, this work emphasises the need to address the social and economic conditions that support cats for effective management.

We find areas that are more densely populated with people tend to harbour more unowned cats. Similar results have been found in terms of colony size^28^ and with unowned cats generally favouring residential areas^9,20^. It is likely that in areas with higher densities of people there are increased resources for unowned cats due a greater likelihood of deliberate food provisioning^9^, increased availability of food waste to exploit^29^ and a rich resource of synanthropic prey species^30–32^. Additionally, given cats are one of the most popular pets^1,2^ the densities of free-ranging owned cats are likely to be increased in these areas, increasing opportunities for breeding and for the pet population to feed into the unowned through abandonment, straying and accidental litters. We further showed links between the socio-economic context of the community and unowned cats, with a positive relationship between unowned cat abundance and indices of deprivation. Similar associations with deprivation have been found with stray cat density in New Zealand^33^. Previous work has recognised lower neutering rates of the unowned^34^ and the owned cats^35^ in deprived areas and that reduced prevalence of neutered cats results in increased feral cat density^15^, indicating that the distribution of unneutered cats is a possible defining feature of unowned cat abundance estimates.

The dependency of unowned cats on human populations in urban areas suggests addressing this as a purely environmental issue is likely to be ineffectual. Our finding of an association with human communities is promising, as it implies additional capacity and targeting to neuter owned and unowned cats can help ameliorate overpopulation of unowned cats. Indeed, cat population management requires considering the potential breeding population that includes both unowned cats and the pet cats that they mix with. Thus, the manner in which the human population manage and care for owned cats is instrumental in mitigating the overpopulation of unowned cats in urban areas. Although neutering rates are generally high, timely neutering to prevent accidental litters is equally important with many owned cats having unplanned litters in the UK^36^. Further work in this area is warranted especially as many cat owners hold misconceptions regarding the age of puberty of cats and are unaware that related cats can mate with each other^36^, which is a particular concern when owners acquire littermates when obtaining new kittens. Additionally, we find strong links between human deprivation and unowned cats, likely mediated by lower rates of neutering of owned and unowned cats within these communities^37,38^. This outcome is likely related to a balance of multiple socio-economic factors and their associated barriers influencing capability, opportunity and motivation^39^ to neuter cats. Regardless of which of these explanations is dominant, extra effort will be required to identify and overcome barriers to prevent overpopulation in deprived areas.

The average density of unowned cats in urban areas across the UK is estimated to be 9.3 cats per km^2^ similar to that of other native carnivores in urban areas (badgers 9.32 per km^2^; foxes; 13.9 per km^2^^40^). However, we find that average values at an area level can range widely from 1.9 to 57 cats per km^2^, indicating localised issues of overpopulation. Whilst there is limited evidence from the UK, some studies of urban unowned cats in other countries have reported much higher densities (Republic of Korea 132–268 cats per km^2^^41^; South Africa 161 cats per km^2^^15^; Puerto Rico 360 cats per km^2^^9^). However, they are largely based on occupied and localised sites, therefore unlikely to be representative of the characteristics of urban areas at broader spatial scales limiting the comparability to this study.

Despite the potential for inaccuracies at finer spatial scales, the spatially explicit quantitative estimates and associated uncertainty of the unowned cat population are critically informative, enabling evidence-based decisions about future allocation of management effort across the UK. Predictions are also likely to become increasingly robust across wider geographic regions, averaging out any inconsistencies as we scale up. Additionally, this framework allows for further improvement and refinement to estimates in future analysis. For example, the discovery of additional model components that explain variation in abundance would further improve estimates.

Although our estimates are based on outcomes of a robustly tested hierarchical modelling approach and our validation indicates no systematic biases, a number of limitations remain. First, the model cannot account for unowned cat abundance beyond the human population densities within our study. In our estimates, we made conservative assumptions regarding areas with a higher human population density than those within the studied regions. Indeed, we would not expect the unowned cat population to increase indefinitely with human population size. Ultimately, cats are likely to reach an eventual carrying capacity based on availability of resources and consequently a plateau in cat numbers. However, our arbitrary threshold for an unrealistic increase in density of unowned cats is conservative and might be exceeded in some places. Conversely, we cannot discount that areas with the highest levels of human habitation may potentially be more inhospitable, with increased risk from anthropogenic impacts and have a lower carrying capacity, such that we may even expect reduced unowned cat numbers at the highest densities. Indeed, cat ownership levels are thought to be lower in London^26^. Whilst our results suggest this is unlikely to significantly alter national estimates, our assumption may cause inaccuracies at a local level. Future efforts to explore the presence and abundance of unowned cats in particularly high-density areas, such as within London, would be valuable. Second, we base our UK estimates on comparable data across countries, with the implicit assumption that our findings are not unique to England. Given the consistency of cat ownership practices and neutering rates across the UK^2,42^, it is likely these comparisons are relevant. However, studies to test this pattern and refine the model further for other countries would be beneficial. Finally, we assume implicitly that unowned density has remained invariant subsequent to data collection. The consistency in the population of the owned cat subgroup over the past decade^1,2^ indicates that wide scale changes in associated groups are unlikely, however our modelling framework provides an opportunity for temporal monitoring of unowned cat populations, which alongside traits of urban areas may also provide a stronger idea of causality. Furthermore, this approach can assess management actions by monitoring localised areas before and after interventions, informing future specific management decisions.

Whilst we provide the first estimate of unowned cats in the UK, we are only able to account for urban areas that make up approximately 13% of total land cover. With the exception of cats on farms, that have access to increased resources, densities of unowned cats in rural areas are anticipated to be much lower, especially where cats live independently from people and rely on hunting for food^22^. Foxes, which have a high degree of dietary overlap with feral cats^43,44^, also are found in much higher densities in urban than rural environments such that the numbers that reside in each are similar despite rural areas forming the majority of the species distribution^40^. We may expect a similar pattern for unowned cats. Indeed, away from human habitation and the associated resources, unowned cat densities will be dependent on prey densities, which may also be mediated by mesopredator competition^45^. Further work quantifying unowned cat numbers in rural areas would be beneficial.

Our work focuses on unowned cats and we are not able to determine the relative proportions that reside in stray and feral subgroups. Although in reality cats are likely to lie on a spectrum of sociability, for management purposes feral cats are largely defined as being unsocialised to humans^46–48^, due to a lack of exposure to people when kittens. Consequently, feral cats are fearful of people and considered less likely to live near human habitation^48,49^. Stray cats are considered lost, or abandoned, previously owned cats and therefore used to human contact. We would therefore anticipate the number of stray cats to be higher in urban areas relative to rural areas, due to increased numbers of owned cats and consequently increased flux between the owned and unowned populations. Indeed, studies in other countries find a high proportion of owned cats are rehomed directly from the stray population^50^, and up to 5% of owned cats go missing potentially moving into the stray population^51,52^. Although research in the UK is limited, with an owned cat population of over 10 million^1,2^ even low levels of uncontrolled feline reproduction, straying and abandonment would contribute to a considerable stray cat population, with over 40,000 known stray cats entering UK shelters every year^3^. Understanding the stray subgroup is especially important as previously owned unneutered cats are able to transition to a feral state within a single generation. It is therefore desirable to evaluate the nature of the relationship between unowned cat sociability and a range of different geographies, with a view to further highlight potential causality and guide management, given the appropriateness of any intervention will depend on whether cats are likely to accept human contact.

Although further model refinement using data from a wider range of geographic areas and with explicit consideration of the unowned cat subgroups should be a priority for future research, we find anthropogenic drivers of unowned cat abundance in urban areas and provide the first evidence-based estimate of unowned cat abundance in the UK. These estimates can be used practically to facilitate rational decision-making. This framework can be used for monitoring at different spatial scales. Additionally, analysing data in real time will allow interventions to be tuned to match the spatial and social characteristics of the population. Our findings suggest that managing the socio-economic influences on unowned cat populations is pivotal to unowned cat management in urban areas.

## Methods

### A framework to estimate unowned cat abundance

In the following sections, we describe the application of an IAM, a hierarchical modelling approach, which estimates unowned cat abundance in discrete geographical units from spatially replicated citizen data, in combination with expert data obtained from 162 sites across five urban areas in England. In doing so, we explored key predictors of unowned cat abundance. We then estimated unowned cat abundance across urban areas in England and the UK with respect to the modelling results. We used WinBUGS^53^ and R^54^ for all data analysis via the R package R2Winbugs^55^ and QGIS^56^ for plotting maps.

### Data collation and preparation

A database of unowned cat count data were compiled from citizen science data and expert data collected throughout a one-year period that began between 2016 and 2018 across five urban areas in the UK. Areas included Beeston, Bradford, Bulwell, Dunstable & Houghton Regis and Everton (Fig. 1). These data were collected as part of Cat Watch, a community partnership project set-up by Cats Protection, a UK feline welfare charity, to control cat numbers^39,57^. Two distinct forms of citizen science data were collected: (1) the first consisted of an initial cross-sectional random-sample door-to-door survey carried out with approximately 10% of households. At that stage, residents were asked how many cats they know of locally and how many they think were owned in the form of a multiple-choice question with the following options; none, 1–2, 3–4, 5–9, 10 or more, from which the number of unowned cats were derived. When a range was selected the central value was taken; for ten or more we used 15 (the average from reports when 10 or more was specified was 14.7). Location data were available for 3101 survey responses, within which there were estimates of 4411 unowned cats; (2) throughout the project, residents were able to report unowned cats in their area directly via social media or through a mobile application. During the study period, 877 reports were received reporting on the locations of 2790 unowned cats. These data were collected according to the study protocol approved by University of Bristol Faculty of Health Science Research Ethics Committee approval number 38661. All methods were performed in accordance with the relevant guidelines and regulations. Informed consent was secured in advance of survey participation. Residents provided report data voluntarily, with no identifying information collected. No experimental protocols were used.

Expert data were obtained from an experienced community team (CT) that recorded when and where an unowned cat was found or confirmed the lack of presence of an unowned cat. The CT carried out extensive door-to-door surveillance across both reported hot spot and cold spot areas. These data are considered of higher quality, due to the ability of the CT to correctly identify an unowned cat and with no risk of double counting the same individual. Unowned cats can be either stray or feral. Protocols to accurately identify a stray cat included; scanning for a microchip, attaching a paper collar to notify potential owners, advertising online, door-to-door notifications, local posters and contacting other animal welfare organisations, including veterinary practices. If no owner was found during this process it was identified as unowned. Feral cats were more likely to be identified via behavioural means; as they have not been socialised to humans, they will be more fearful and will not approach humans^47^. If they have already been neutered they may also have their left ear “tipped”. During the study period, there were 601 records from the CT, reporting on the location of 605 confirmed unowned cats. All three of these data sources provided detailed location data (postcodes and/or addresses) enabling geo-referencing of unowned cat location data.

To account for duplicate sightings, the citizen science data required clustering to account for neighbours in close-proximity reporting the same cats. There is limited understanding of urban unowned cats in the UK, however studies of urban unowned cats in other areas indicate home range sizes between 3.7 and 10.4 ha for urban areas^58,59^. Studies on unowned cats in the UK indicate that home ranges vary between 10 and 15 hectares^60^. We assume a maximum 20 ha home range, equivalent to a circular area with a diameter of 504 m. Consequently, we apply a 500 m cluster function in R that derives clusters of cat sightings that are within 500 m of each other. The individual records were maintained as replicate counts within each cluster. Clustering of 500 m has also been shown to provide reasonable estimates in an urban area with high expert coverage (91%), where you would not anticipate cat numbers to be significantly inflated above those observed by experts^25^. In the absence of expert data, the effect of violating this assumption (i.e. reporting them as replicate sightings when they are not) would result in lower estimates of cats. However, where expert data is available, the effect of violating this assumption would result in bias in the observation parameters, not estimates of the cats themselves, which are also inferred from the expert data that do not contain duplicate sightings.

### Data analysis

We applied an integrated abundance model (IAM) within a Bayesian framework that combines count data across sites from two forms of citizen science data and expert data^25^. The hierarchical structure of the IAM enables it to borrow strength from the sites with expert data to inform detection biases of citizen science data, including detection probability of an unowned cat and false positives due to misidentification of an owned cat as unowned. The goal of the inference is to estimate the abundance of unowned cats within each site and explore covariates as predictors of population density.

Specifically, observed citizen science counts at each site *i* and during each replicate survey *j* are linked to true site-specific population sizes (*N*_*i*_) via a detection probability (p) and the expected number of misidentifications (*m*). We apply a Poisson distribution to account for additional stochasticity in spatial replicates not accounted for in the systematic biases (*m* and *p*). Each type of citizen science data is modelled separately to account for the different biases in collection methods between the survey data (y) and report data (u):

$$ {y_{i,j}}\sim {\text{ Poisson }}({N_i}{p_y} + {m_y}) $$

$$ {u_{i,j}}\sim {\text{ Poisson }}({N_i}{p_u} + {m_u}) $$

Expert consensus (w_i_) was available on the abundance of individuals for 104 sites and linked to true population sizes via a Poisson observation error.

$$ {w_i}\sim {\text{ Poisson }}\left( {N_i} \right) $$

We additionally assume that where expert counts are available they are accurate at the level of presence or absence.

$$ {z_i}\sim {\text{Bernoulli}}\left( \Omega \right) $$

$$ {N_i}_= {z_i}{\lambda_i} $$

whereby z_i_ is a binary measure of occurrence, with each of the *i* sites occupied or not, that is modelled as a Bernoulli random variable determined by occupancy probability (Ω). True site-specific population sizes (*N*_*i*_) are therefore a function of whether a site is occupied or not and a site-specific mean *λ*_*i*_*.* When expert data on occurrence can be inferred from expert consensus this was included in z_i_.

We extend the original development of an IAM^25^ described above to model the log the site specific mean (*λ*_*i*_) as a linear function of covariates (*x*) using the following linear relationship:

$$ log{\lambda }_{i} = \mu +\sum_{j=1}^{n}{\beta }_{j}\;{x}_{j,i}+{\varepsilon }_{i}$$

$$\varepsilon \sim N(0,{\sigma }^{2})$$

where *x*_*j*,* I*_ are the values of the *j*th covariate across sites *i*, βs are the regression coefficients for each covariate and ɛ is the residual site-specific variation providing estimates of unexplained variance. We also fitted a model without covariate effects to gain an estimate of total site-specific variance. The proportional reduction in the residual site-specific variation component is a measure for the proportion of the site-specific variance in abundance explained by that covariate or covariates.

To assess the credibility of covariate effects we calculated the probability that their effects were positive [*P*(β > 0)] or negative [*P*(β < 0)].

We used priors for each parameter as follows: uniform distributions U(0,1) for detection probability and occupancy; uniform distributions (0,5) for misidentification parameter; normal distribution N(0,100) for mu, uniform distribution (0,3) for standard deviation of the random effect. Preliminary simulations were assessed for convergence of the chains by visually checking mixing of the chains and more formally using the Brooks–Gelman–Rubin criterion ($$ \overset{\lower0.5em\hbox{$\smash{\scriptscriptstyle\frown}$}}{r} $$^61^). Following the initial trials for each simulation we ran three chains of 20 000 with a burn‐in of 10,000 for each analysis, yielding a sample size of 30,000 iterations, from which full posteriors were stored. The IAM that included quintiles as a covariate took longer to converge, necessitating a longer model run of 100,000, a burn-in of 60,000 and retained every 4th value to yield a similar sample of 30,000 iterations.

### Validation of IAM model

To check the validity of our inference with specific focus on our regression coefficients that are subsequently used to predict population sizes we used six approaches to model diagnostics.

First, we simulated datasets structured according to the raw data and parameterised using the model estimates to check model performance under similar scenarios and with the presence of covariates. We explore performance in terms of accuracy (proportion of simulations that capture the true value in their credible intervals) and bias (tendency for posterior distributions to lie above or below true values).

Second, we performed general Bayesian model-checking procedures including convergence of MCMC chains, which were assessed using the Gelman–Rubin statistic and values less than 1.1 were interpreted as indicating convergence. Additionally, we compared a null model, with just a random effects term, with a model containing the covariates. In this way, remaining variances after accounting for covariates were known. We calculated the proportion of variance explained by the covariates when modelled independently and combined.

Third, influence of variation across study sites on model fit for regression parameters was assessed using five-fold cross-validation where the model was refitted five times, each time withholding all data from one study site until all sites had been held out once.

Fourth, to assess the influence of the different types of citizen science data on the regression parameter we re-ran the analyses separately to contain just one type of citizen science data (survey IAM and report IAM) alongside the expert data. Only sites where data were available were included, this amounted to 157 sites in the survey IAM (five sites were excluded) and 134 sites in the report IAM (28 sites were included). The sites included in the survey IAM spanned the full range of IMD deciles (1–10) and human population densities (238–15,129 people per km^2^). Whereas, the sites in the report IAM only spanned the full range of IMD deciles (1–10), with population densities not incorporating the lowest and the highest density areas (390–13 847 people per km^2^).

Fifth, we assessed how prior specifications for regression parameters influenced the results. We varied the parameter range between 10 magnitudes lower to two magnitudes higher than the original by refitting the IAM with three different prior distributions for the regression parameters, the original (B ~ uniform (−5, 5)), a first alternative with a more narrow distribution (B ~ uniform (−0.5, 0.5)) and a second alternative with a wider distribution (B ~ uniform (−10, 10)).

Finally, we tested drivers of unowned cat abundance by fitting generalised linear models with logarithmic link and quasi-Poisson error that has known significance testing procedures.

### Quantification of unowned cat abundance in England

We estimated the unowned cat population in the urban areas of England, accounting for uncertainty in our estimates, by multiplying data-derived posterior distributions of regression coefficients by deciles of deprivation and standardised human population density from urban areas in England. The calculation may be expressed as:

$$ {\text{Unowned}}\;{\text{cat}}\;{\text{abundance}} = {\text{exp}}(\mu \, + \, {\beta_{\text{IMD deciles}}}*{\text{IMD }} + {\beta_{\text{human pop dens}}}*{\text{PD}}) \, *{\text{ SP}} $$

where μ is the posterior distribution of the intercept from the IAM, β_IMD deciles_ and β_human pop dens_, are the posterior distributions of the regression parameters of IMD and standardised population density respectively, and IMD and PD are the true deprivation and human density values for the area, as sourced from the ONS (see supplementary table 2). Additionally, we incorporate a scaling parameter (SP), which is the area of the site relative to the area in the study.

At the smallest geographic unit we focussed our estimates on ONS defined lower Super Output Areas (LSOAs), which are the small area geographies for England designed to be socially homogeneous with a relatively even population size with 1500 residents on average, but they vary in their area size and consequently population density. All urban LSOAs in England (defined as towns, cities and conurbations with more than 10,000 residents according to Communities and Local Government guidelines) were classified into decile groups according to the level of deprivation and assigned human population density, which were standardised. Using this approach, we predicted the abundance and density (per km^2^) of unowned cat populations for 27,246 urban LSOA’s in England. The total unowned cat population was calculated as a summation across all sites. For each calculation we used the full posterior distributions from the IAM output, in doing so we maintain proper estimates of uncertainty in our predictions and present the mean values and 95% credible intervals for all results.

### Quantification of unowned cat abundance in UK

We applied the same calculation described above to predict the number of unowned cats across urban areas in the UK. However, small-area statistical geographies differ between countries in the UK, defined by their respective national statistics agencies. Lower layer super output areas (LSOAs) are defined in England and Wales, super output areas (SOAs) in Northern Ireland and data zones in Scotland. LSOAs have populations of around 1500 people, while SOAs are slightly larger with typical populations of around 2000 people. Scottish data zones are smaller with populations of 500 to 1000 people.

Urban–Rural classifications differ across different countries in the UK. Consequently, using the ONS definition, we only included data zones in Scotland and super output areas in N. Ireland as urban areas if they were part of settlements of 10,000 people. This equated to 530 out of 890 SOA in Northern Ireland and 4909 out of 6976 data zones in Scotland. These were combined with 28,549 urban LSOA in England and Wales. Population densities for these areas were sourced from national statistics agencies (Supplementary Table 2).

Measures of socioeconomic deprivation differ across country borders in the UK and naively comparing across these measures can result in problems in analysis^27^. In the absence of a consistent IMD decile across countries, we apply a previously published consistent measure of deprivation, in the form of adjusted IMD scores^27^, based on 2011 indices. These quintiles of deprivation provided the means to inform on unowned cat abundance across the UK.

### Validation of predictions

First, we tested the influence of different model assumptions on national population estimates. This included: (1) the influence of London on estimates; (2) the inclusion of areas with high population density and (3) the influence of the different measures of deprivation (Deciles vs. Quintiles) on England estimates, where both data are available. Second, we test the impact of using outcomes from the GLM approach vs. the IAM. Finally, in the absence of any prior estimates of unowned cats in these regions we tested the validity of population predictions by determining whether cat population density derived from predictions were comparable with those derived from the original IAM analysis of the raw data. We compared the overlap of credible intervals and correlation for cat density at three different scales: (1) across 44 LSOA where there was also spatial coverage of the raw data; (2) at the scale of the study town or city and (3) the average density across all study sites.

## Supplementary Information

Supplementary Information.

Source Data.

## Data Availability

Unowned cat predictions are available in the source data file, along with the posterior distributions of coefficients used to calculate them. The raw cat sightings data are part of an ongoing project collated and managed by Cats Protection. Data can be available from the corresponding author upon reasonable request subject to the access conditions of Cats Protection. Code to simulate example datasets are included as supplementary material. Links and citations for all freely available predictor data sets are available in Supplementary Table 2. The R script for simulating abundance data with and without the inclusion of covariates, and the two corresponding forms of the IAM for analysing the data are available in the supplementary files.
