# Supplementary material for: Human influences shape the first spatially explicit national estimate of urban unowned cat abundance
Source: Sci Rep. 2021 Oct 28;11:20216. doi: 10.1038/s41598-021-99298-6 (PMC8553937; doi:10.1038/s41598-021-99298-6)
Supplement: Supplementary file 1 — Supplementary Information. [file 41598_2021_99298_MOESM1_ESM.pdf]

Supplementary Material

Supplementary Table 1. Summary of area characteristics and associations with unowned cat abundance from a GLM. *indicates significant *P* value. [a]run in lieu of deciles

Characteristic	Mean (range) or proportion in each group for factors	Test statistic	*P* value
IMD decile	3 (1-10)	24.65	<0.001*
Human population density (km^2)	4903 (238-15129)	58.02	<0.001*
Proportion population white	0.81 (0.17-0.98)	1.81	0.18
Proportion population Asian	0.10 (0-0.75)	0.60	0.44
Dominant housing type	Flat (49%)<br>Detached House (6%)<br>Semi-detached house (22%)<br>Terraced house (23%)	0.69	0.56
Proportion of households that are flats	0.21 (0-0.92)	0.21	0.64
Urban settlement type	Urban city and town (24%)<br>Urban major conurbation (36%)<br>Urban minor conurbation (40%)	2.01	0.14
IMD quintile[a]	5 (1-5)	36.77	<0.001*

Supplementary Table 2. Key Resources Table. Datasets used in this work are freely available from the relevant national statistics agencies.

Data	Source	Description	Identifier
Lower layer Super Output Area population density (National Statistics)	ONS	Mid-year (30 June) population density of Lower layer Super Output Areas (LSOAs) in England and Wales based on estimates of the usual resident population	https://www.ons.gov.uk/peoplepopulationandcommunity/populationandmigration/populationestimates/datasets/lowersuperoutputareapopulationdensity
English Indices of Deprivation	Ministry of Housing, Communities & Local Government	This dataset contains a range of measures which form the	https://opendatacommunities.org/resource?uri

2019 - LSOA Level		Indices of Deprivation 2019 at LSOA level.	=http%3A%2F%2Fopendatacommunities.org%2Fdata%2Fsocietal-wellbeing%2Fimd2019%2Findices
Rural Urban Classification (2011) of Lower Layer Super Output Areas in England and Wales	ONS	The 2011 rural-urban classification (RUC) of lower layer super output areas in England and Wales is based on the 2011 RUC of output areas published in August 2013, and allows users to create a rural/urban view of LSOA level products.	https://data.gov.uk/dataset/b1165cea-2655-4cf7-bf22-dfbd3cdeb242/rural-urban-classification-2011-of-lower-layer-super-output-areas-in-england-and-wales
2014 Property type	VOA	2014 data of properties by property type for LSOA11	https://data.london.gov.uk/dataset/property-build-period-lsoa
2011 Census ethnicity data	ONS	2011 ethnicity census data	https://data.gov.uk/dataset/c16c372d-81fc-4f42-9010-361fb0662533/ethnic-group
Settlement 2015 Documentation	NISRA	Lookup table of the super output areas to urban rural classification and 2011 population census	https://www.nisra.gov.uk/publications/settlement-2015-documentation
Urban Rural Classification	Scottish Government	Urban Rural classification for data zones Scotland	https://statistics.gov.scot/data/urban-rural-classification
Scotland population census	Scotland census	Usual resident population for Data Zones 2011	https://www.scotlandscensus.gov.uk/
Scotland area data zones	Scottish Government	Land Area (based on 2011 Data Zones)	https://statistics.gov.scot/data/land-area-2011-

			data-zone-based
Ireland pop size	NISRA	Usual resident population for SOAs 2011 census	https://www.ninis2.nisra.gov.uk/public/Home.aspx
Ireland area	NISRA	Area size for SOA	https://www.ninis2.nisra.gov.uk/public/Home.aspx
UK quintiles of deprivation	Abel, Barclay & Payne	Published adjusted IMD scores that allow consistent analysis across the UK (reference 27)	University of Bristol data.bris Research Data Repository, https://data.bris.ac.uk/data/, DOI: 10.5523/bris.1ef3q32gybk001v77c1ifmty7x.

Supplementary Table 3. Comparison of unowned cat population estimates in urban areas in England calculated with different underlying assumption. Values in bold are those reported in the main text

Modelling approach regression coefficients derived from	Assumptions in predictions	Mean unowned cat Population	Lower 95% Credible Interval	Upper 95% Credible interval
**IAM**	**Unowned cat density does not increase beyond human population limits of the study**	**179,494**	**118,391**	**258,882**
GLM	Unowned cat density does not increase beyond human population limits of the raw data	165,093	NA	NA

IAM	Unowned cat density does not increase beyond human population limits of the study Excluding London from analysis	163,568	107,793	236,892
IAM	Unowned cat density does increase with human population density beyond limits of the raw data	198,468	118,716	331,360

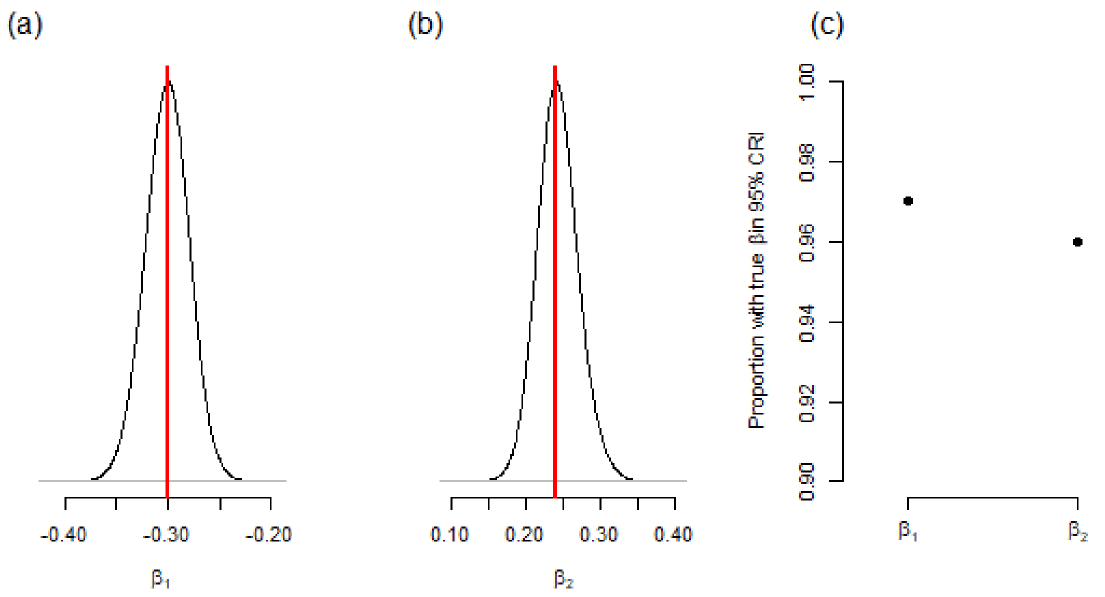

Supplementary Figure 1. Accuracy and bias of regression parameters from model simulations with known covariate effect sizes (β_1=-0.30; β_2=0.24) in a scenario modelled to reflect the underlying data in the field system. (a-b) There were no observed bias in the posterior distributions for the regression coefficients. c) both regression coefficients had high accuracy (>95%). Bias is observed as the full posteriors from all simulations. Accuracy is measured here by the proportion of simulations where the true value is captured by the 95% CRI.

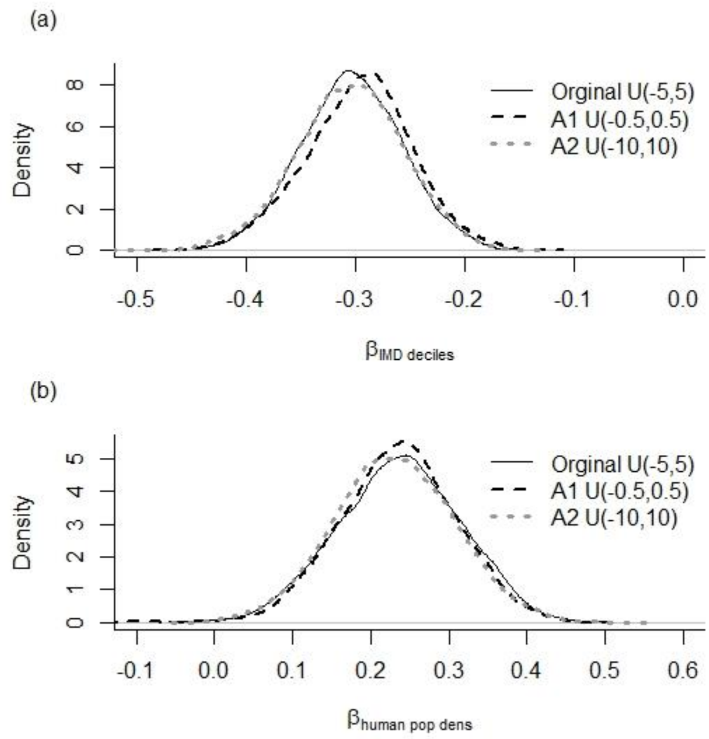

Supplementary Figure 2. Posterior density plots for original and alternative priors for (a) IMD deciles and (b) human population density as a predictor of unowned cat abundance

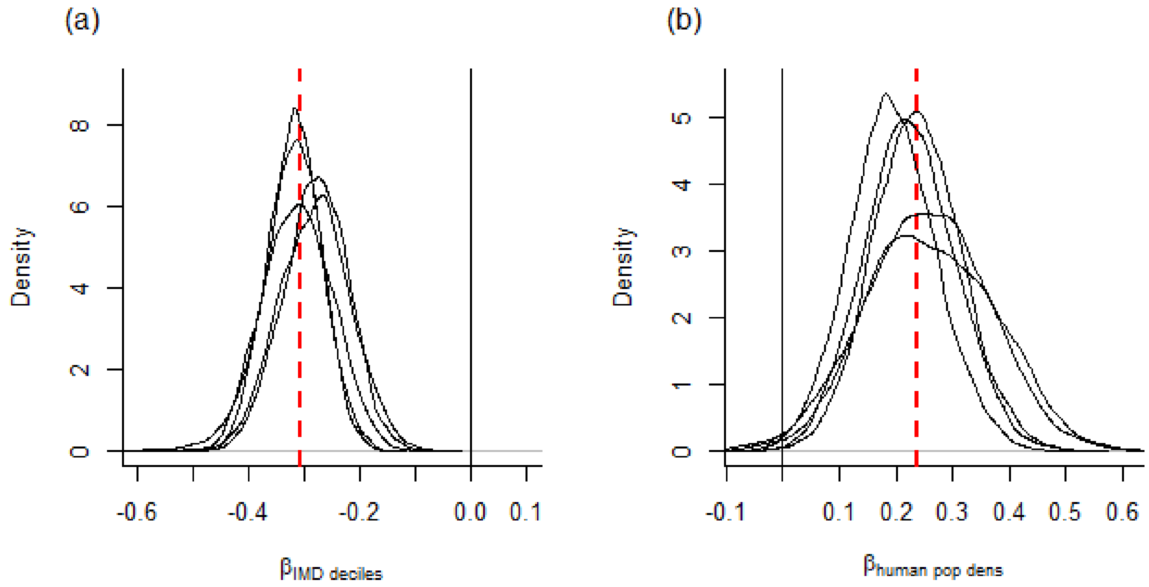

Supplementary Figure 3. Study area-specific cross-validation of regression coefficients for (a) IMD deciles and (b) human population density. Cross-validation analysis was carried out by re-calculating effect size with stepwise removal of each urban area (n=5) and re-analysis. The resulting posterior distributions of effect sizes overlapped. Mean value from the global model shown by dashed red line.

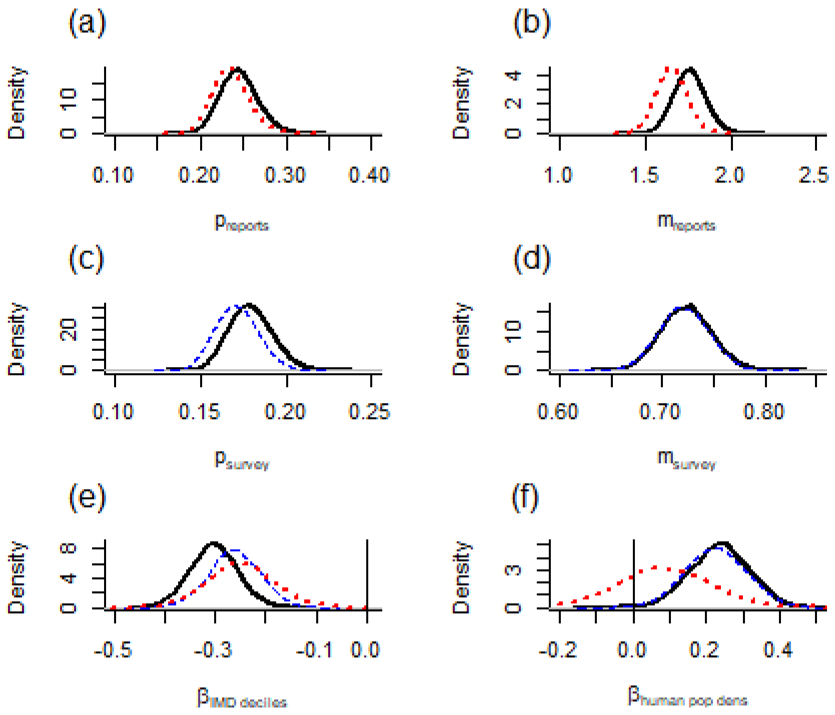

Supplementary Figure 4. Posterior distributions of detection parameters (a-d) and the effect sizes for (e) IMD deciles and (f) human population densities for the full model containing both reports and survey data for 162 sites (black solid line), a reduced model with just survey data for 157 sites (blue dashed line) and a reduced model with just report data for 134 sites (red dashed line). Whilst the reduced models overlapped with the full model, the notable difference was the weaker effect size for human population density in the report IAM as a result of sites not covering the full range of population densities in the full model. Therefore, reports alone did not offer the spatial coverage to determine the effect of population density.

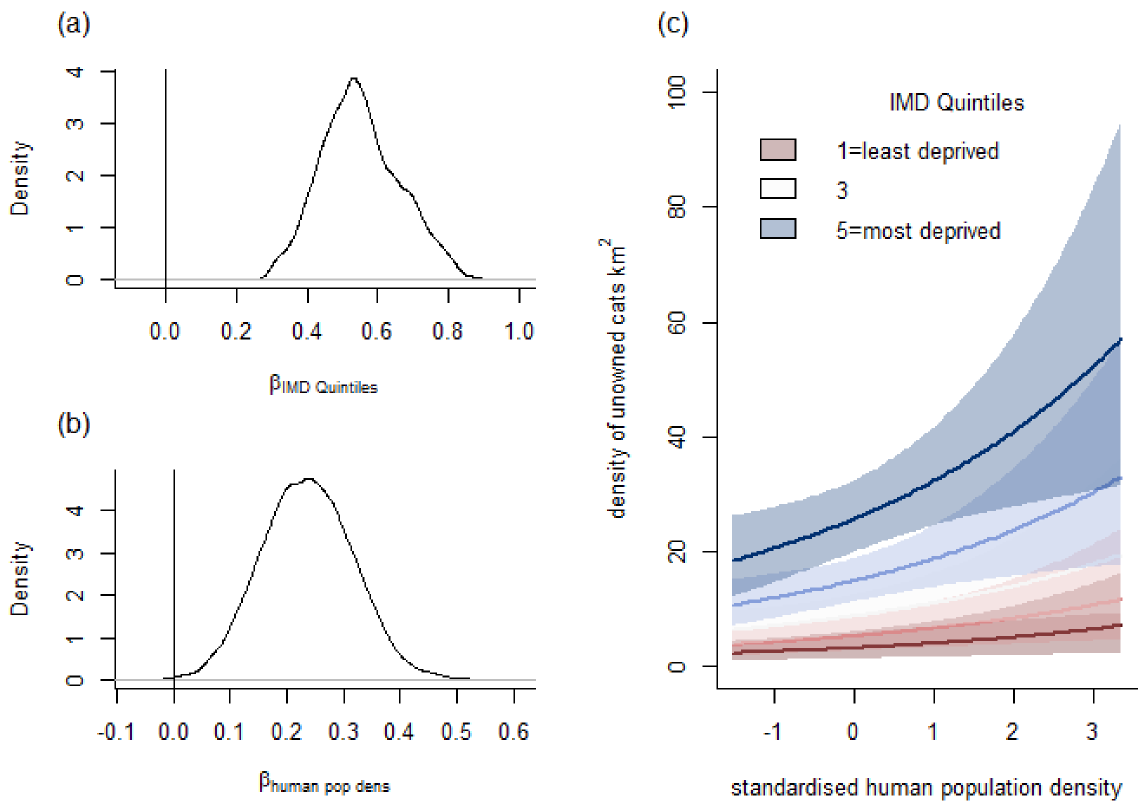

Supplementary Fig 5. The influence of IMD quintiles and standardised human population density on unowned cat abundance. a-b) show the corresponding posterior distributions of the regression coefficients from an IAM c) showing the predicted relationship and the corresponding 95% CRI between density of unowned cats and covariates

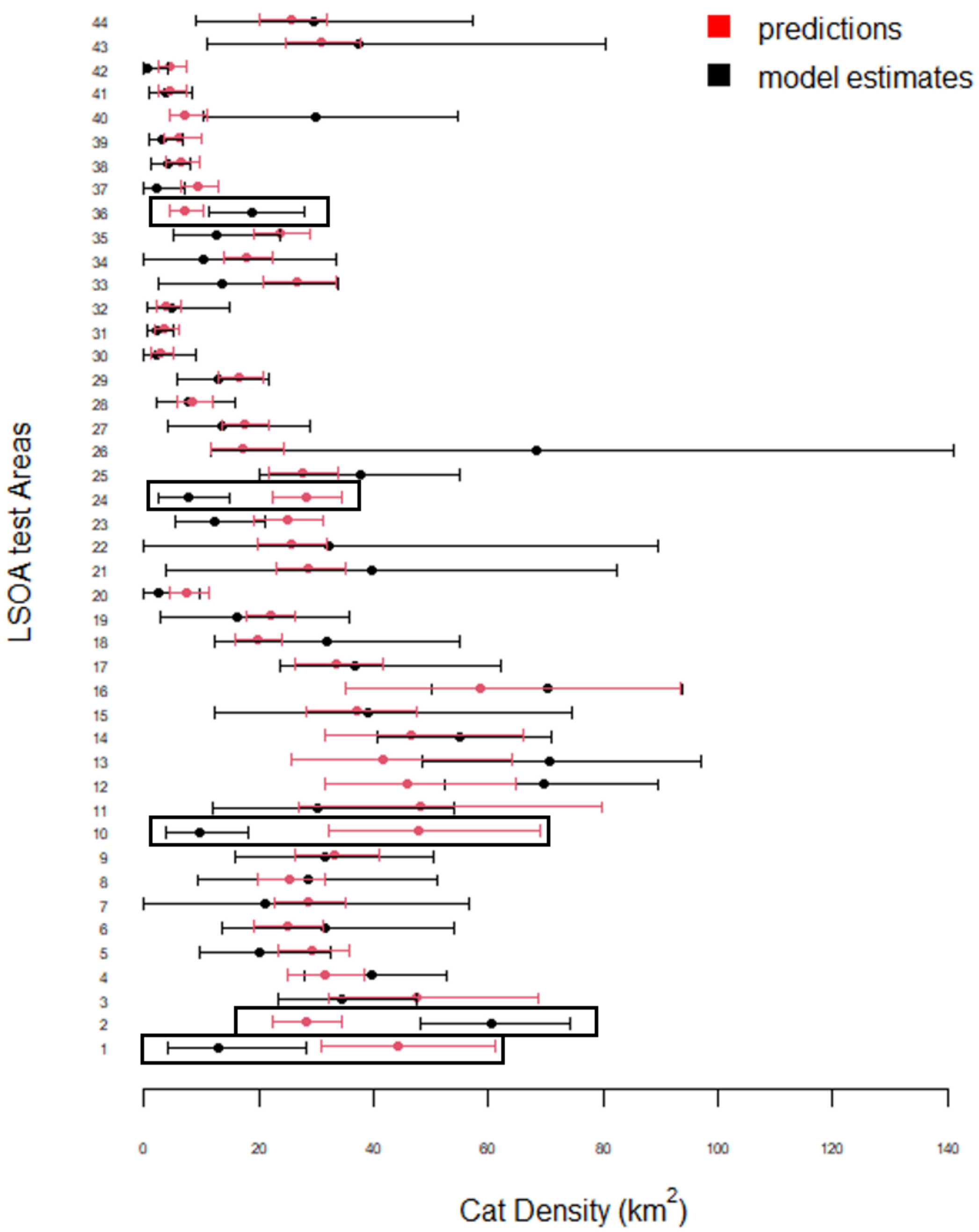

Supplementary Figure 6. Model validation across 44 LSOA in England that were covered by the raw data collection. Outputs from the original IAM analysis are compared with predictions. 89% of predictions (n=39/44) overlapped the 95% credible intervals for density estimates modelled from the raw data. The five areas that show no overlap between 95% CRIs are outlined. The mean model estimates and predictions were significantly correlated with no evidence of systematic biases in the predictions (i.e. consistent over- or under-estimation)

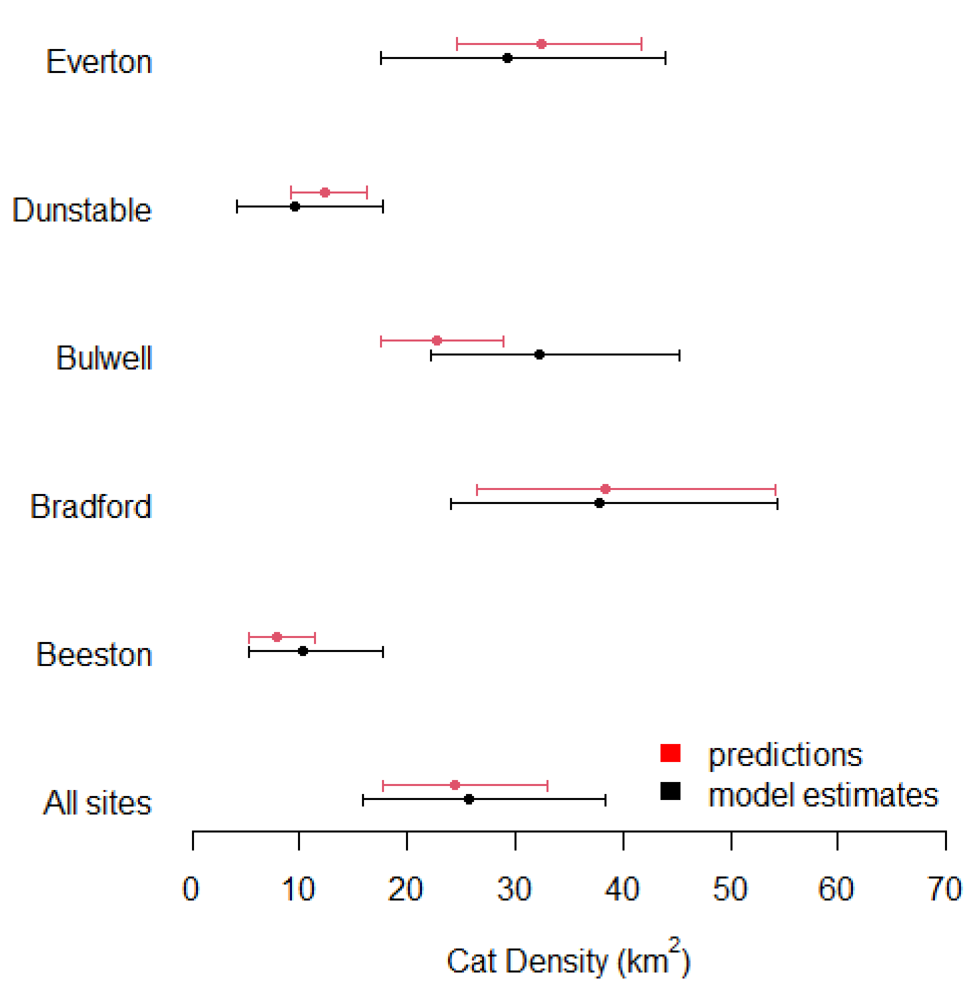

Supplementary Figure 7. Model validation across the five study areas in England and a combined measure across all five sites ("All sites") that were covered by the raw data collection. Outputs from the original IAM analysis are compared with predictions. 100% of predictions overlapped the 95% credible intervals for density estimates modelled from the raw data. There were no evidence of systematic biases in the predictions (i.e. consistent over- or under- estimation)

Supplementary Code

```
## Human influences shape the first spatially explicit national estimate of urban unowned cat abundance

## McDonald and Skillings

## 2021

####################################################################################################
```

##This file contains the complete core of the R code used to simulate data with the same structure as the raw data and described in the paper under "Validation of IAM model"

##We simulate data under scenarios with and without covariates and correspondingly include two forms of the IAM, which are described in the accompanying paper (McDonald and Skillings)

These simulations represent our field system of interest, fuller descriptions of IAMs and adaptable code are available in McDonald and Hodgson, 2021 as referenced in the paper.

##

##Specifically, the code shows how to simulate data comparable to that of our study system, with one version incorporating auxiliary data as covariates.

##The input data simulated include survey data, report data and expert data. Model code for IAM both with and without covariates are also included.

##For more general data simulations and IAM model code please refer to an earlier paper McDonald and Hodgson, 2021

```
#########################################################################
#########################################################################
##########


#########################################################################
#########################################################################
##########
```

Simulate data with two forms of citizen science data, one form of expert data with same structure as data used in this study without covariates

```
#########################################################################
#########################################################################
##########
```

nsites=162 #number of sites

#number of survey responses per site

```
Ry<-c(0	,9	,38	,57	,66	,25	,0	,4	,35	,0	,0	,0
	,46	,12	,54	,0	,12	,58	,23	,10	,20	,13	,0	,47
	,30	,23	,23	,0	,1	,0	,4	,1	,

47	,26	,9	,3	,0	,4	,0	,54	,63	,0	,1	,13	,48
	,16	,44	,44	,41	,44	,35	,29	,37	,57	,26	,0	,4
	,42	,5	,11	,48	,33	,11	,18	,

5	,6	,8	,3	,24	,25	,26	,6	,2	,21	,105	,12	,15
	,7	,9	,5	,34	,2	,11	,5	,3	,19	,11	,44	,25
	,18	,23	,9	,21	,5	,19	,9	,
```

```
14	,22	,1	,11	,41	,9	,2	,1	,35	,5	,13	,17	,14
	,33	,26	,31	,32	,45	,18	,10	,25	,38	,14	,25	,1
	,14	,3	,15	,15	,0	,13	,38	,

36	,34	,12	,28	,28	,32	,25	,0	,1	,19	,1	,14	,28
	,5	,27	,3	,1	,7	,34	,2	,18	,28	,23	,11	,1
	,27	,24	,26	,23	,9	,6	,3	,23	,0	)

#number of reports per site

Ru<-c(8		,19	,9	,29	,13	,20	,6	,16	,4	,4	,12	,1
	,19	,8	,22	,1	,7	,4	,6	,3	,1	,2	,1	,3
	,5	,7	,7	,1	,2	,3	,4	,

0	,0	,0	,0	,0	,14	,14	,19	,5	,2	,1	,5	,8
	,5	,2	,12	,5	,3	,2	,0	,3	,0	,0	,0	,2
	,0	,1	,0	,0	,0	,0	,0	,

0	,21	,25	,10	,37	,25	,4	,3	,24	,20	,12	,2	,10
	,3	,2	,1	,2	,1	,1	,0	,0	,0	,0	,0	,10
	,4	,4	,20	,10	,1	,7	,17	,11	,2	,2	,1	,2
	,54	,6	,3	,4	,4	,4	,2	,3	,

14	,6	,6	,1	,1	,1	,1	,1	,2	,0	,0	,0	,0
	,0	,0	,0	,0	,0	,3	,16	,20	,22	,2	,6	,10
	,2	,2	,10	,5	,4	,4	,1	,5	,2	,2	,4	,0
	,0	,0	,0	,0	,0	,0	,0	,0	,0	,0	,0	,0
	,0	,0	,0	,0	,0)#number of reports per site


#specify observation error for citizen science data

p=0.18 #detection probability-survey

m=1 #misidentification of non-target species per site -survey

p2=0.24 #detection probability-report

m2=2 #misidentification of non-target species per site -report


#create arrays for survey data y and report data u

y=array(dim=c(nsites,max(Ry)))

mu=array(dim=c(nsites,max(Ry)))

u=array(dim=c(nsites,max(Ru)))

muu=array(dim=c(nsites,max(Ru)))
```

```
z<-rep(1,162)#create starting vector of binary occurance measure

#simulate heterogeneity across sites which is comparable to that of raw data but with no covariate drivers

totalN=640 #total abundance across sites

n<-rmultinom(n=1,size=totalN,prob=c(5.312379367 ,18.58066667 ,11.10358773
        ,13.4761526 ,3.746474767 ,11.58939003 ,3.550668707 ,0 ,0
        ,1.47738381 ,22.38558167 ,1.595121955 ,5.2072659 ,1.744932825
        ,8.593770067 ,3.29394092 ,0 ,14.14955327 ,1.151140311 ,0
        ,2.73229381 ,1.393648206 ,1.643125529 ,1.4575866 ,2.180009603
        ,6.121225133 ,16.01603667 ,4.663120517 ,1.10047573 ,5.320330357 ,0
        ,1.428968109 ,

0.340853902 ,0.008843361 ,5.1331234 ,0.502362609 ,3.311828367 ,4.505239533
        ,2.047732 ,0.637718068 ,0.898273724 ,0 ,0 ,8.207518267
        ,0.737024491 ,2.388777309 ,0 ,0 ,1.030386324 ,0 ,0 ,0 ,0
        ,0 ,0 ,0 ,0 ,0 ,0 ,0.087150568 ,0.026796649
        ,0.124398256 ,3.109285748 ,0.218650078 ,7.121396533 ,10.6029693 ,7.5278312
        ,3.803746653 ,12.4712131 ,3.733684273 ,

6.456253333 ,11.20858437 ,1.370626233 ,13.6145103 ,6.1973377 ,13.5721149
        ,5.848801243 ,18.2230196 ,1.496510821 ,0.526518642 ,4.465435007
        ,4.778177803 ,6.119363597 ,0.24349982 ,0.498223677 ,13.4348563
        ,5.019928857 ,3.987177867 ,0.53275528 ,3.753606547 ,2.466560407
        ,0.248274094 ,0 ,1.135902712 ,1.171160107 ,1.98519822 ,0
        ,1.77822952 ,2.217045487 ,0 ,0.906426198 ,2.23012898 ,

0 ,2.568186893 ,0.778158502 ,5.86006666 ,0 ,1.858495293 ,4.144690477
        ,2.552005173 ,3.68414486 ,0.6025769 ,0 ,0 ,0.661621809
        ,1.323355308 ,0 ,0 ,0 ,0 ,0 ,0 ,0 ,0 ,0 ,0
        ,2.782137323 ,11.79175773 ,12.113594 ,3.322237767 ,11.07964937 ,6.448633
        ,7.650196867 ,7.585989967 ,4.56954985 ,3.3872176 ,5.91809849
        ,3.66556709 ,

1.84533695 ,6.473985767 ,3.848298797 ,5.989414707 ,3.32117159 ,0
        ,1.688500845 ,12.06287983 ,2.922587583 ,0 ,0 ,7.0673398 ,8.001546
        ,0.141345784 ,27.28822963 ,7.5670681 ,5.381398267 ,9.167708233 ,5.3707059
        ,0.260253662 ,3.515467444 ,27.5635563 ,7.448827467 ,0 ))

z[n==0]<-0

#Simulate expert data subject to observation error (w)

w=rep(0,nsites)
```

```
w=rpois(nsites,n)# model observation error in expert counts


remove<-sample(1:nsites,58,replace=FALSE)# randomly remove expert counts from 58 sites to replicate a site coverage of 64%

w64<-w

w64[remove]<-NA

#Remove from occupancy also

z64<-z

z64[remove]<-NA

#Simulate survey data subject to detection, misidentification and Poisson error (y)

   for(i in 1:length(Ry[Ry>0])){

    mu[Ry>0,][i,] <- (n[Ry>0][i]*p)+m

    y[Ry>0,][i,1:Ry[Ry>0][i]]<-rpois(Ry[Ry>0][i],lambda=mu[Ry>0,][i,1:Ry[Ry>0][i]])

  }

#Simulate report data subject to detection, misidentification and Poisson error (y)

 for(i in 1:length(Ru[Ru>0])){

    muu[Ru>0,][i,] <- (n[Ru>0][i]*p2)+m2

    u[Ru>0,][i,1:Ru[Ru>0][i]]<-rpois(Ru[Ru>0][i],lambda=muu[Ru>0,][i,1:Ru[Ru>0][i]])

  }


################################################################################################################################################################
##########

## Simulate data with two forms of citizen science data, one form of expert data with covariates using same structure as data used in paper

################################################################################################################################################################
##########

nsites=162 #number of sites

#number of survey responses per site

Ry<-c(0     ,9    ,38   ,57   ,66   ,25   ,0    ,4    ,35   ,0    ,0    ,0
      ,46   ,12   ,54   ,0    ,12   ,58   ,23   ,10   ,20   ,13   ,0    ,47
      ,30   ,23   ,23   ,0    ,1    ,0    ,4    ,1    ,
```

```
47	,26	,9	,3	,0	,4	,0	,54	,63	,0	,1	,13	,48
	,16	,44	,44	,41	,44	,35	,29	,37	,57	,26	,0	,4
	,42	,5	,11	,48	,33	,11	,18	,

5	,6	,8	,3	,24	,25	,26	,6	,2	,21	,105	,12	,15
	,7	,9	,5	,34	,2	,11	,5	,3	,19	,11	,44	,25
	,18	,23	,9	,21	,5	,19	,9	,

14	,22	,1	,11	,41	,9	,2	,1	,35	,5	,13	,17	,14
	,33	,26	,31	,32	,45	,18	,10	,25	,38	,14	,25	,1
	,14	,3	,15	,15	,0	,13	,38	,

36	,34	,12	,28	,28	,32	,25	,0	,1	,19	,1	,14	,28
	,5	,27	,3	,1	,7	,34	,2	,18	,28	,23	,11	,1
	,27	,24	,26	,23	,9	,6	,3	,23	,0	)

#number of reports per site

Ru<-c(8		,19	,9	,29	,13	,20	,6	,16	,4	,4	,12	,1
	,19	,8	,22	,1	,7	,4	,6	,3	,1	,2	,1	,3
	,5	,7	,7	,1	,2	,3	,4	,

0	,0	,0	,0	,0	,14	,14	,19	,5	,2	,1	,5	,8
	,5	,2	,12	,5	,3	,2	,0	,3	,0	,0	,0	,2
	,0	,1	,0	,0	,0	,0	,0	,

0	,21	,25	,10	,37	,25	,4	,3	,24	,20	,12	,2	,10
	,3	,2	,1	,2	,1	,1	,0	,0	,0	,0	,0	,10
	,4	,4	,20	,10	,1	,7	,17	,11	,2	,2	,1	,2
	,54	,6	,3	,4	,4	,4	,2	,3	,

14	,6	,6	,1	,1	,1	,1	,1	,2	,0	,0	,0	,0
	,0	,0	,0	,0	,0	,3	,16	,20	,22	,2	,6	,10
	,2	,2	,10	,5	,4	,4	,1	,5	,2	,2	,4	,0
	,0	,0	,0	,0	,0	,0	,0	,0	,0	,0	,0	,0
	,0	,0	,0	,0	,0)#number of reports per site


#specify observation error for citizen science data

p=0.18 #detection probability-survey

m=1 #misidentification of non-target species per site -survey

p2=0.24 #detection probability-report

m2=2 #misidentification of non-target species per site -report


#create arrays for survey data y and report data u

y=array(dim=c(nsites,max(Ry)))

mu=array(dim=c(nsites,max(Ry)))
```

```
u=array(dim=c(nsites,max(Ru)))

muu=array(dim=c(nsites,max(Ru)))


###simulate covariate values

###IMD Deciles

IMD<-(sample(1:10, 162, replace=T))

#Human population density (scaled)

PopDens<-runif(n=162,min=-1.530681,max=3.355628)

#Specify effect sizes

beta1=-0.3

beta2=0.24

#simulate site-specific abundance according to covariate effects

n<-exp(2.4+(beta1*IMD)+(beta2*PopDens))

z<-rep(1,162)


#Simulate expert data subject to observation error (w)

w=rep(0,nsites)

w=rpois(nsites,n)# model observation error in expert counts

z[w==0]<-0


remove<-sample(1:nsites,58,replace=FALSE)# randomly remove expert counts from 58
sites to replicate a site coverage of 64%

w64<-w

w64[remove]<-NA

#Remove from occupancy also

z64<-z

z64[remove]<-NA

#Simulate survey data subject to detection, misidentification and Poisson error (y)

 for(i in 1:length(Ry[Ry>0])){

    mu[Ry>0,][i,] <- (n[Ry>0][i]*p)+m

    y[Ry>0,][i,1:Ry[Ry>0][i]]<-rpois(Ry[Ry>0][i],lambda=mu[Ry>0,][i,1:Ry[Ry>0][i]])
```

```
  }
#Simulate report data subject to detection, misidentification and Poisson error (y)
 for(i in 1:length(Ru[Ru>0])){
    muu[Ru>0,][i,] <- (n[Ru>0][i]*p2)+m2
    u[Ru>0,][i,1:Ru[Ru>0][i]]<-rpois(Ru[Ru>0][i],lambda=muu[Ru>0,][i,1:Ru[Ru>0][i]])
  }


##############################################################################
##############################################################################
##########
## IAM model without covariates but with random effects - for a more general version of IAM see an earlier paper McDonald and Hodgson, 2021
##############################################################################
##############################################################################
##########
# Load necessary library
library(R2WinBUGS)
# Path where WinBUGS is located (might be different on your machine)
bugs.dir <- "C:/WinBUGS14"


# IAM
sink("IAM.txt")
cat("
    model {
    # Priors
    omega~dunif(0,1) #occupancy
    p~dunif(0,1) #detection survey
    p2~dunif(0,1) #detection reports
    m~dunif(0,5)#misidentification survey
    m2~dunif(0,5)#misidentification reports
    mu~dnorm(0,0.01)
    tau.lam<-1/(sd.lam*sd.lam)
```

```
    sd.lam~dunif(0,3)
    #Likelihood
    #observation model expert counts
    for (i in 1:nsites){ #Loop over R sites
    log(lambda[i])<-mu+epsilon[i]
    epsilon[i]~dnorm(0,tau.lam)
    z[i]~dbern(omega)
    N[i]<-z[i]*lambda[i]
    W[i]~dpois(N[i])
    #Observation model for replicated amateur counts
    for (j in 1:R){
    y[i,j]~dpois(Y[i,j])
    Y[i,j]<-(N[i]*p)+m
    }
    for (j in 1:P){
    u[i,j]~dpois(U[i,j])
    U[i,j]<-(N[i]*p2)+m2
    }


    }
    ##derived quantities
    totalN<-sum(N[]) }
    ",fill=TRUE)
sink()

win.data<-list(y=y, W=w64, R=ncol(y), nsites=nrow(y),z=z64,u=u, P=ncol(u))
params<-c( "p","p2","totalN","m","m2","omega","mu","sd.lam")
ni=20000
nt=1
nb=10000
```

nc=3

#Model can run with initials not specified however, including initials can prevent winbugs trap messages in some scenarios e.g.

inits<-function(){list(omega=runif(1,0,1),p=runif(1,0,1),p2=runif(1,0,1),m=sample(1,1,5))}

out1<-bugs(win.data,inits,params,"IAM.txt",n.chains=nc,n.thin=nt,n.iter=ni,n.burnin=nb,debug=TRUE,bugs.directory = bugs.dir, working.directory = getwd())

out1$summary

##

IAM model with covariates and random effects - for a more general version of IAM see an earlier paper McDonald and Hodgson, 2021

##

Load necessary library

library(R2WinBUGS)

Path where WinBUGS is located (might be different on your machine)

bugs.dir <- "C:/WinBUGS14"

sink("IAM_covariates.txt")

cat("

model {

Priors

omega~dunif(0,1) #occupancy

p~dunif(0,1) #detection survey

p2~dunif(0,1) #detection reports

m~dunif(0,5)#misidentification survey

m2~dunif(0,5)#misidentification reports

mu~dnorm(0,0.1)

beta~dunif(-5,5)

beta2~dunif(-5,5)

```
    tau.lam<-1/(sd.lam*sd.lam)
    sd.lam~dunif(0,3)
    #Likelihood
    #observation model expert counts
    for (i in 1:nsites){ #Loop over R sites
    log(lambda[i])<-mu+beta*D[i]+beta2*H[i]+epsilon[i]
    epsilon[i]~dnorm(0,tau.lam)
    z[i]~dbern(omega)
    N[i]<-z[i]*lambda[i]
    W[i]~dpois(N[i])
    #Observation model for replicated amateur counts
    for (j in 1:R){
    y[i,j]~dpois(Y[i,j])
    Y[i,j]<-(N[i]*p)+m
    }
    for (j in 1:P){
    u[i,j]~dpois(U[i,j])
    U[i,j]<-(N[i]*p2)+m2
    }


    }
    ##derived quantities
    totalN<-sum(N[])
   }
    ",fill=TRUE)
sink()

#####has employment in at moment
win.data<-list(y=y, W=w64, R=ncol(y), nsites=nrow(y),z=z64,u=u,
P=ncol(u),D=IMD,H=PopDens)
```

```
params<-c( "p","p2","totalN","m","m2","omega","beta","beta2","mu","sd.lam")

ni=30000

nt=1

nb=20000

nc=3

#Model runs with initials not specified however, including initials can prevent winbugs trap messages in some scenarios e.g.

inits<-function(){list(omega=runif(1,0,1),beta=runif(1,-1,1),p2=runif(1,0,1),m=sample(1,1,5))}

out2<-
bugs(win.data,inits,params,"IAM_covariates.txt",n.chains=nc,n.thin=nt,n.iter=ni,n.burnin=nb,
debug=TRUE,bugs.directory = bugs.dir, working.directory = getwd())

out2$summary
```
